# Reply to: Long-term psychological outcome after discharge from intensive care

**DOI:** 10.5935/0103-507X.20190023

**Published:** 2019

**Authors:** Sara Pereira, Teresa Cardoso, Sara Cavaco

**Affiliations:** Intensive Care Unit, Hospital Santo António, Centro Hospitalar do Porto - Porto, Portugal.

**To the Editor**

We have read the letter sent by Dr. Fernanda Lima-Setta el al.^(1)^ with great interest and very carefully, and we thank you in advance for your consideration.

We agree that the importance of posthospitalization syndrome in intensive care is undeniable and that the article "Long-term psychological outcomes after discharge from intensive care" represents a pilot study, which will be further developed in the future and in which we intend to increase the sample size and draw solid conclusions.^(2)^ Considering the previously described limitations of this study, we do not intend to lead professionals to change their decisions or to interfere with clinical practice. Our intent is to draw the attention of the community working in intensive care medicine to the relevance of this topic and to its consequences.

Fortunately, medicine has evolved to recognize the patient at risk and prevent disease, and it is also important to follow this path with regard to this syndrome. The chosen methodology included the study of the probability of developing cognitive deficit, whether or not each of the chosen variables was present, by simple logistic regression; thus, the odds ratio in table 3^(3)^ is crude and not adjusted. As Lima-Setta et al.^(1)^ have emphasized, considering the limited number of patients, progression of the study with multivariate logistic regression would not be adequate.

Limitations of the study were clearly described in the article, drawing attention to the limited number of patients included and to the absence of evaluation of the functional, cognitive and psychological status prior to admission and discharge from the intensive care unit, which would allow assessing the impact of hospitalization in a more objective manner.

Loss to follow-up is a limitation that was partially overcome by the lack of significant differences between the two groups with regard to demographic and clinical data.

As mentioned in the discussion, the association found between hypoxia and a lower cognitive impact contradicted the known pathophysiology. The possible explanation is that patients who suffered more hypoxia belonged to a younger group, with lower comorbidities. Being young and having lower comorbidities are protective factors against cognitive decline, not hypoxia, and these two factors explain the difference found.

The most relevant result of our study was the finding that throughout the five years after discharge from the intensive care unit, there was a progressive improvement in postadmission syndrome in intensive care units, especially with regard to the cognitive domain, psychological domain (anxiety, depression and posttraumatic stress) and quality of life domain, which suggests long-term reversibility of this syndrome.

With this pilot study, we intend to reveal the existence of postadmission syndrome in intensive care units, encouraging the study of associated risk factors to plan for its prevention and of the long-term impact to better define the prognosis for patients and their relatives.

*Sara Pereira* *Intensive Care Unit, Hospital Santo
António, Centro Hospitalar do Porto - Porto,
Portugal.* *Teresa
Cardoso* *Intensive Care Unit, Hospital Santo
António, Centro Hospitalar do Porto - Porto,
Portugal.* *Sara Cavaco* *Intensive
Care Unit, Hospital Santo António, Centro Hospitalar do Porto - Porto,
Portugal.*
